# Directed Self-Assembly on Photo-Crosslinked Polystyrene Sub-Layers: Nanopattern Uniformity and Orientation

**DOI:** 10.3390/ma9080648

**Published:** 2016-08-01

**Authors:** Haeng-Deog Koh, Mi-Jeong Kim

**Affiliations:** Inorganic Materials Laboratory, Samsung Advanced Institute of Technology (SAIT), Samsung Material Research Complex, 130 Samsung-ro, Yeongtong-gu, Suwon-si, Gyeonggi-do 443-803, Korea; hd.koh@samsung.com

**Keywords:** block copolymer, directed self-assembly, polystyrene sub-layer, photo-crosslinking, DSA uniformity

## Abstract

A photo-crosslinked polystyrene (PS) thin film is investigated as a potential guiding sub-layer for polystyrene-block-poly (methyl methacrylate) block copolymer (BCP) cylindrical nanopattern formation via topographic directed self-assembly (DSA). When compared to a non-crosslinked PS brush sub-layer, the photo-crosslinked PS sub-layer provided longer correlation lengths of the BCP nanostructure, resulting in a highly uniform DSA nanopattern with a low number of BCP dislocation defects. Depending on the thickness of the sub-layer used, parallel or orthogonal orientations of DSA nanopattern arrays were obtained that covered the entire surface of patterned Si substrates, including both trench and mesa regions. The design of DSA sub-layers and guide patterns, such as hardening the sub-layer by photo-crosslinking, nano-structuring on mesas, the relation between trench/mesa width, and BCP equilibrium period, were explored with a view to developing defect-reduced DSA lithography technology.

## 1. Introduction

The self-assembly of block copolymers (BCPs) has, over the past decade, become a powerful technique for fabricating functional templates and scaffolds for advanced devices such as bit-patterned media [1], memory devices [2], nanowire-based transistors [3,4], arrays of quantum dots or metallic nanoparticles [5,6], and ultrafiltration membranes [7]. This has been made possible by the intrinsic ability of BCPs to form highly ordered polymeric structures with periodicities on a nanometer scale. Recent developments in directed self-assembly (DSA) lithography with cylindrical and lamellar BCP phases have presented a new possibility for next-generation industrial nanolithographic patterning tools [8,9,10]. In order to fabricate defect-reduced nanopatterns, several environmental forces have been adapted to direct the self-assembly of structures through solvent annealing [11,12], thermal annealing [13,14], electromagnetic fields [15], shear forces [16], or sub patterns produced either topographically [17,18,19] or chemically [20,21]. With all kinds of DSA, it is essential to understand the role played by the interfaces that exist beneath the BCP layers.

The grafting of polymers onto a substrate (i.e., the use of polymer brushes) has been used to modify the wettability of their surface. Jung et al. reported that cylinder orientations of polystyrene-block-poly (dimethylsiloxane) (PS-*b*-PDMS) BCP were more uniform on PDMS-brushed Si topographical patterns than on PS-brushed ones [22]. Furthermore, Kim et al. have demonstrated that a PS homopolymer brush, the thickness of which is dependent on the molecular weight of PS, is more useful for tuning the surfaces energy of substrates than the more widely-used grafting of poly (styrene-r-methyl methacrylate) (PS-*r*-PMMA) random copolymer with hydroxy-terminated functional moieties [23]. This means that vertically orientated nanostructures of cylinder- and lamella-forming polystyrene-block-poly (methyl methacrylate) (PS-*b*-PMMA) BCP can be formed using a specific range of interfacial energy-matched brush thicknesses. Polymer brushes, however, are severely limited in their ability to provide the necessary control over monolayer thickness, which is essential to achieving the long-range uniformity needed during DSA lithography for wafer-scale industrial semiconductor production. The spin-coated brush layer generally consists of a monolayer grafted on top of a substrate and a multilayer that is physically adsorbed on the top, simply washing the unanchored upper layer with a solvent can produce defects in the residual multilayer part owing to its intermixing with the BCP layers. This, in turn, can result in non-uniform self-assembled layers.

It was recently reported by Li et al. that irradiation with deep UV radiation in doses of ~20 J/cm^2^ induces full crosslinking in polymer backbone chains due to the formation of new C-C bonds [24]. Similar photo-crosslinking reactions have been widely used to selectively harden the PS domain of PS-based self-assembled BCP nanostructures such as honeycombs and opal structures, as well or for fabricating micron-scaled lithographic patterns for use in various applications [25,26,27]. Herein, a photo-crosslinked PS sub-layer film prepared by ultraviolet (UV) irradiation of thermally annealed hydroxy-terminated PS (PS-OH) films of varying thickness is used for the first time to control the orientation of BCP cylinders during their DSA on a topographically patterned substrate. The sub-layer consisted of a vinyl polymer with an aromatic group side chain, which can be fully cross-linked by irradiation with deep UV light; and a terminal hydroxy group grafted onto the native Si surface of the topographically patterned substrate. The thickness of the photo-crosslinked PS film could be easily tuned by controlling the initial thickness of the as-spun PS-OH film before photo-crosslinking. The use of this sub-layer provides thickness-dependent interfacial energy tunability resulting in defect-reduced DSA nanostructures, and provides a simple way to control the parallel and orthogonal orientation of BCP cylinder arrays relative to the Si substrate.

## 2. Results and Discussion

### 2.1. Photo-Crosslinked PS Sub-Layers

**Figure 1:**
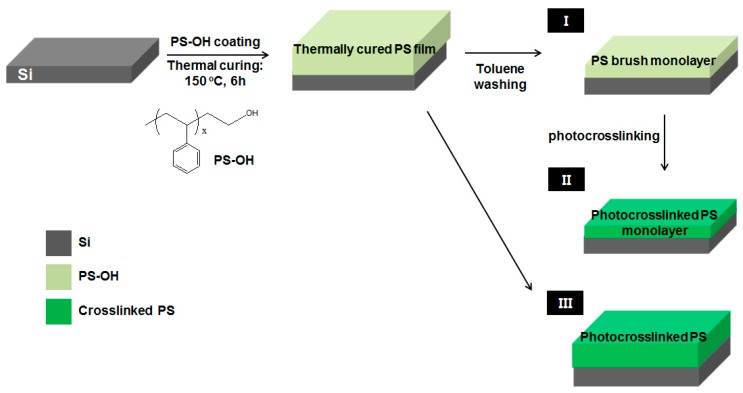
Process for preparing three kinds of PS sub-layer: PS-OH polymer is spin-coated onto an Si substrate and thermally cured; (**I**) PS brush monolayer is formed after washing with toluene to remove any unanchored PS-OH moieties; (**II**) photo-crosslinked PS monolayer and (**III**) thick photo-crosslinked PS film are obtained after UV irradiation.

The process used to create the photo-crosslinked PS sub-layer films is depicted in Figure 1, wherein a PS-OH film is first spin coated onto an Si wafer, and then thermally cured at 150 °C under vacuum to chemically anchor the PS-OH polymer chains via dehydration. Washing this thermally cured, 61.5 nm-thick PS multilayer film with toluene to remove any unanchored PS-OH moieties produced a PS brush monolayer (I) with a thickness of 16.7 nm. The PS monolayer and PS multilayer film were then irradiated in air at room temperature with 20 J/cm^2^ of UV light (wavelength: 254 nm) to form fully photo-crosslinked PS thin films (II, III). The type-II film had a thickness of 12.9 nm, making it about 23% thinner than the type-I film. This was due to the polymer main chains becoming more densely packed as a result of new C-C bonds being formed [24]. The photo-crosslinked surface was smooth and shiny, with a golden color. The surface energies of the films were determined by measuring their contact angles, which revealed that increasing the UV radiation from 3 to 20 J/cm^2^ caused the photo-crosslinked PS film to become more hydrophilic. Thus, its surface energy can be tuned from 43.5–48.3 mN/m simply by altering the irradiation energy density used to create it (Figure S1). The surface energy modification is likely a result of hydrophilic carbonyl groups being formed as side products during the photo-crosslinking reaction [24]. In addition, the surface energy of the Si substrate was 55 J/cm^2^; therefore, all PS sublayers provide hydrophobic surfaces compared with the Si substrates.

Unlike the two-step process used to form type-II PS monolayers, the direct UV irradiation of the thermally cured PS multilayer film without washing it in toluene resulted in the formation of a significantly thicker (40.3 nm) type-III photo-crosslinked PS film. This simplified reaction was advantageous in that it allowed the thickness of the photo-crosslinked PS multilayered films to be controlled more easily from 27.8 to 61.5 nm by changing the spin-coating speed or concentration of the PS-OH solution (Figure S2). In contrast, the thickness of the type-II film could only be controlled by varying the molecular weight of the polymer used. Nevertheless, both photo-crosslinking processes were capable of forming hardened PS sub-layers suitable for BCP spin coating and thermal annealing, as no intermixing was observed between the upper BCP layer and sub-layer. The denser packing of the type-III film meant that it was 35%–40% thinner than the thermally cured PS film. In addition, it did not undergo any further changes in thickness when subsequently washed with toluene, indicating that all of its polymer chains had been fully crosslinked and stabilized.

**Figure 2:**
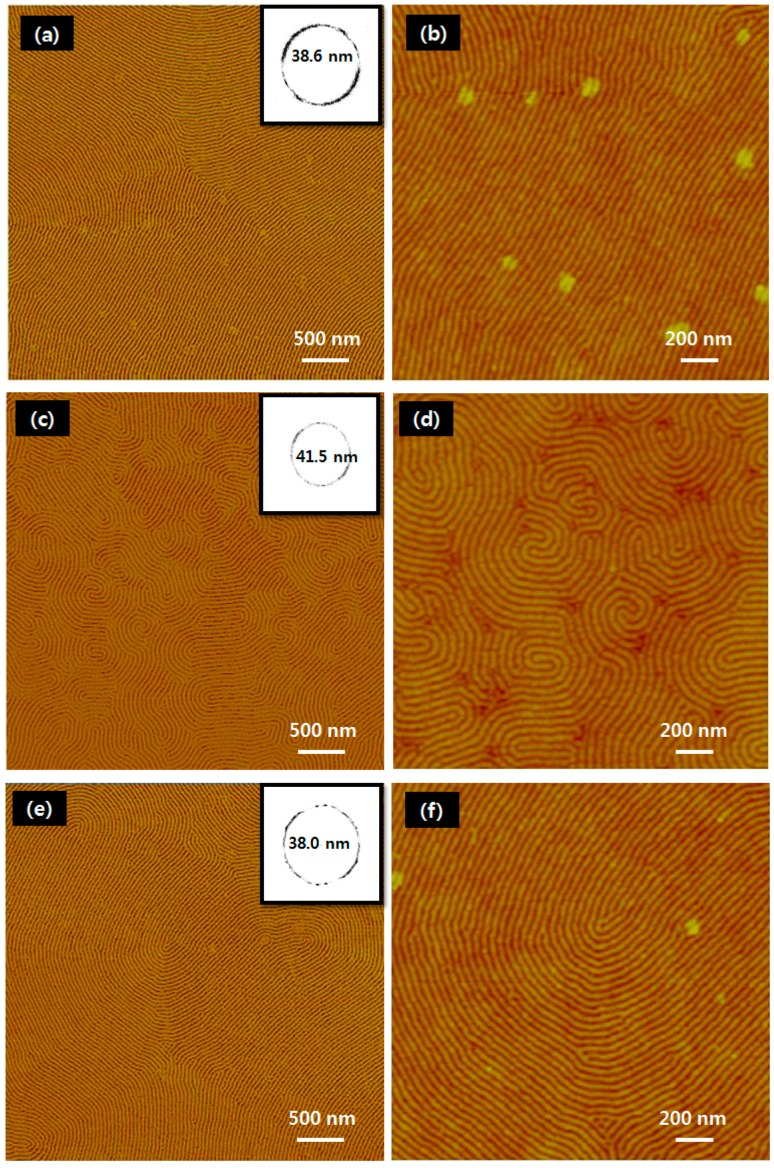
Sub-layer-dependent morphology of PS-*b*-PMMA cylinder arrays and their equilibrium period (L_0_), as measured via FFT analysis of AFM images. Microphase separation of BCP on (**a**,**b**) native Si reference substrate; (**c**,**d**) PS brush monolayer (I) and (**e**,**f**) photo-crosslinked PS film (II). All BCP films were thermally annealed at 280 °C for 60 min.

Figure 2 shows the self-assembled monolayers of cylinder-forming PS-*b*-PMMA BCP (with PMMA as the minor block) that were formed on the non-brushed Si substrate (Figure 2a,b), non-crosslinked PS brush monolayer film I (Figure 2c,d), and photo-crosslinked type-II PS film (Figure 2e,f). These BCP films were thermally annealed at 280 °C for 60 min in a nitrogen atmosphere, giving a final thickness of 30 nm that is ideal for the formation of half-cylinder structures [28]. The equilibrium period (L_0_) and correlation length were determined for each type of sub-layer using fast Fourier transform (FFT) analysis of a low-magnification atomic force microscopy (AFM) image. The L_0_ value of 38.6 nm obtained for the BCP formed on a native Si surface (Figure 2a, inset) was less than the 41.5 nm of the non-crosslinked PS brush monolayer (Figure 2c, inset), but comparable to the 38.0 nm of the photo-crosslinked PS layer (Figure 2e, inset). The BCP correlation length for the type-II film was about 1–2 μm (Figure 2f), which is quite similar to that of the BCP film on the native Si surface (Figure 2b), but much greater than the few hundred nanometers of the type-I film (Figure 2d). Harrison et al. have reported that the correlation length for arrays of polystyrene-block-poly(butadiene) (PS-*b*-PB) cylinders on a native Si wafer is slightly greater than that for similar arrays on a PS brush-treated substrate due to the different degrees of surface lubrication, as well as the pinning effect [29]. These results therefore suggest that in the case of cylinder-forming PS-*b*-PMMA BCP (with PMMA as the minor block), the formation of arrays with high correlation lengths is more likely on hydrophilic native Si substrates and photo-crosslinked PS surfaces than on relatively hydrophobic surfaces such as those covered with PS brush monolayers. Note that increasing the correlation length of BCP cylinders is desirable because it can result in a decrease in BCP topological defects.

### 2.2. Topographical DSA on Photo-Crosslinked PS Sub-Layers

**Figure 3:**
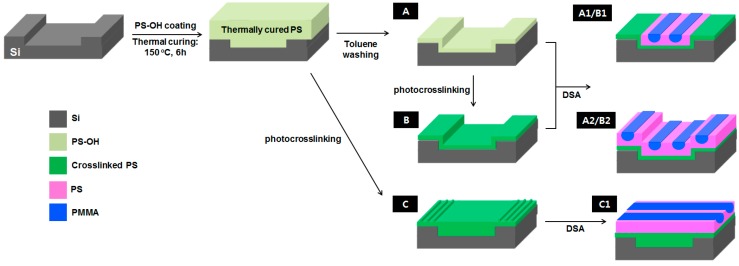
Procedure for forming PS-*b*-PMMA DSA cylinder arrays (A1/B1, A2/B2, C1) on different sub-patterns: (**A**) PS brush monolayer; (**B**) photo-crosslinked PS monolayer; and (**C**) photo-crosslinked PS. Two different types of parallel DSA array (A1 and A2, or B1 and B2) were formed depending on BCP thickness, while an orthogonal DSA array (C1) was formed on sub-pattern C by PS grooves generated in mesa regions. The Si trench depth was 30 nm.

Figure 3 illustrates the topographically directed self-assembly of PS-*b*-PMMA BCP using three sub-patterns (A–C) prepared using the same procedure as the type I-III sub-layers (Figure 1), except that an Si trench pattern was used as the substrate. The uniformity of DSA on different sub-layer types (A vs. B) and its orientation with different thicknesses of photo-crosslinked sub-pattern (B vs. C) were investigated, the results of which are also listed in Figure 3. With sub-pattern C, groove patterns of photo-crosslinked PS were generated on the mesa regions, while the trench domains were covered by a flat photo-crosslinked PS film. This may be the result of the thermally cured PS film on the mesa and trench regions undergoing different degrees of shrinkage during the photo-crosslinking process. The DSA of B1 and B2 on sub-pattern B (and A1 and A2 on sub-pattern A) is orientated parallel to the trench edge, whereas the DSA of C1 has an orthogonal orientation over sub-pattern C.

**Figure 4:**
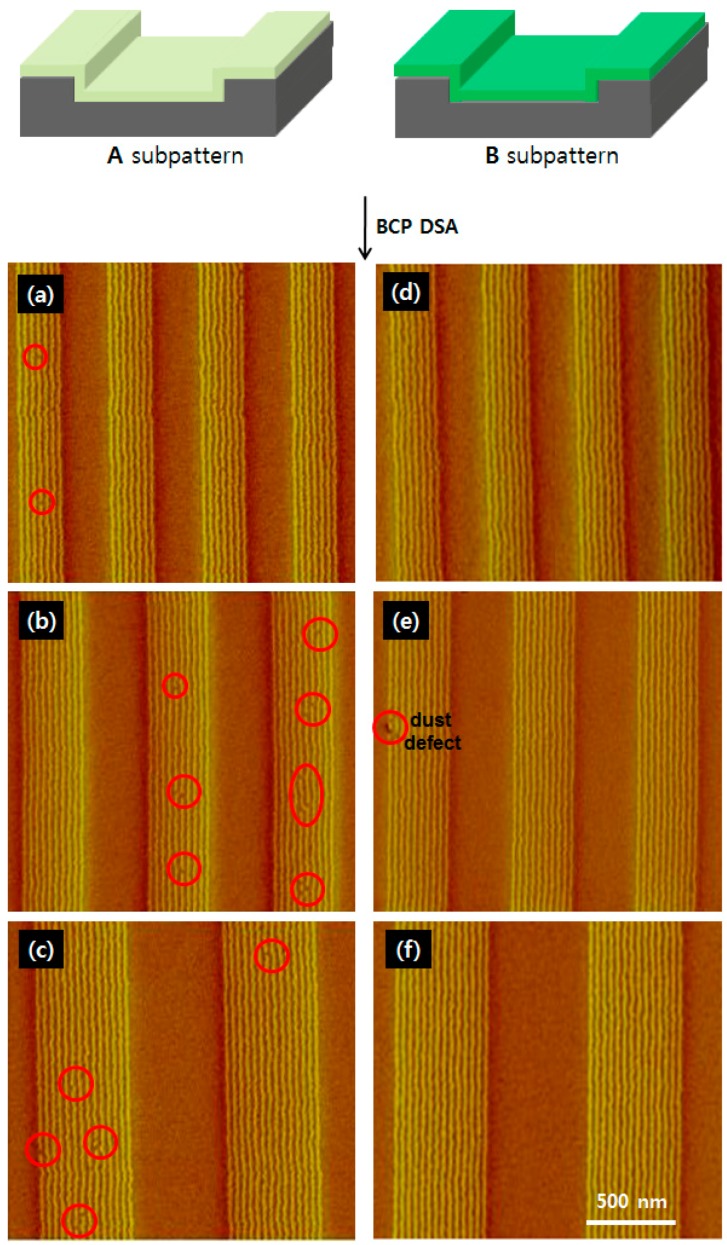
AFM phase images of PS-*b*-PMMA cylinder arrays on (**a**–**c**) non-crosslinked PS brush monolayers on topographically patterned Si substrate patterns (sub-pattern A); and (**d**–**f**) photo-crosslinked PS films (sub-pattern B). Trench widths were (**a**,**d**) 280, (**b**,**e**) 380 and (**c**,**f**) 530 nm. The BCP thickness in the trench is 15 ± 1 nm.

The uniformity of PS-*b*-PMMA cylinders A1 and B1 produced parallel to two differently prepared monolayer sub-patterns A and B by DSA can be seen by comparing their number of defects in Figure 4, which include dislocations and line failures in the 280, 380 and 530 nm-wide trenches. After the BCP films were thermally annealed at 280 °C for 60 min, the surface topographies of the cylinder arrays were imaged via AFM in phase mode without being subjected to etching. This found that most of the PMMA cylinders were oriented parallel to the direction of the trenches. As summarized in Table 1, the calculated values for the confined period (L_c_) were the same for sub-patterns A and B, but varied depending on the trench width: 40.0 nm with a trench width of 280 nm (seven lines; Figure 4a,d), 38.0 nm with a trench width of 380 nm (10 lines; Figure 4b,e) and 37.8 nm with a trench width of 530 nm (14 lines; Figure 4c,f). The L_c_ value of A1 DSA arrays on sub-pattern A (37.8–40.0 nm) was lower than the equilibrium L_0_ value of 41.5 nm (Figure 2c) when the arrays were formed on an unpatterned Si surface, regardless of the trench width. With B1 arrays on the photo-crosslinked sub-pattern B, on the other hand, the L_c_ value was much closer to the L_0_ value at 38.0 nm (Figure 2e). This L_c_ value of 38 nm was the same as that obtained with a trench width of 380 nm (Figure 4e); however, the L_c_ value increased to 40.0 nm with a trench width of 280 nm (Figure 4d), and decreased slightly to 37.8 nm with a trench width of 530 nm (Figure 4f). These results are in keeping with the groove widths [30,31]. Though no significant BCP dislocation defects were observed in either of the two sub-layers with a trench width of 280 nm, wider trenches (380 and 530 nm) produced numerous BCP defects in the arrays formed on sub-pattern A, yet the arrays on sub-pattern B maintained their uniformity. This indicates that defect-reduced DSA can be achieved by precisely designing and fabricating the trench width so that it is a multiple of the equilibrium period of BCP. When considered along with the fact that cylinders with longer correlation lengths on a photo-crosslinked sub-layer (Figure 2f) are more likely to form on a photo-crosslinked sub-layer of PS than on a non-crosslinked PS brush monolayer (Figure 2d), it is evident that preventing intermixing between the upper BCP layer and photo-crosslinked PS sub-layer can help suppress dislocation defects and line failures.

**Figure 5:**
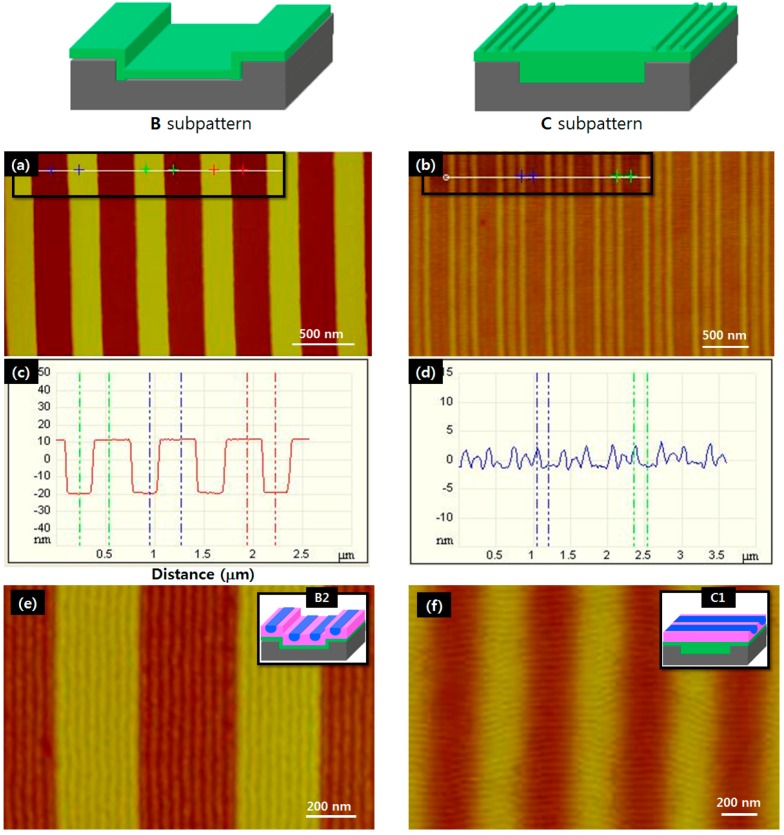
(**a**,**b**) AFM topographic images and (**c**,**d**) height-profiles of photo-crosslinked PS sub-patterns. Sub-pattern B was formed by photo-crosslinking a PS monolayer after removing residual PS multilayers, while sub-pattern C was formed through direct photo-crosslinking of a thick PS multilayer; (**e**,**f**) AFM images of (**e**) parallel B2 and (**b**) orthogonal C1 DSA of PS-*b*-PMMA cylinders on photo-crosslinked sub-patterns B and C, respectively.

Figure 5a,b show AFM topographic images of sub-patterns B and C. The bottom-to-top height of sub-pattern B (~30 nm) in Figure 5c was the same as the inner depth of the Si trenches before coating, indicating that a uniform thickness of photo-crosslinked PS film was formed on the mesa regions and in the trenches. The structure of sub-pattern C was confirmed from its AFM image and depth profiles (Figure 5b,d). The photo-crosslinked PS groove pattern on the mesa regions was in the form of three uniform strips parallel to the topographic pattern on the Si substrate, with grooves that were ~120 nm wide and ~3 nm deep. The photo-crosslinked PS film that filled the trenches was flat and had a roughness of ~1 nm.

When a BCP film coating <25 nm thick over the mesa regions of the sub-layers was thermally annealed, no residual film fragments were observed in the mesa regions. This suggests that the entire mass of BCP mass migrated from the mesa regions into the trenches due to the dewetting phenomena. In contrast, a thicker BCP film (>30 nm on mesa regions) remained stable on the mesa regions, suggesting that no dewetting-induced migration of the BCP occurred. Thus, by depositing a BCP film thicker than 30 nm, single-layered arrays of PS-*b*-PMMA cylinders can be expected to form over the entire surface area of a topographically patterned substrate. As shown in Figure 5e, this produces well-defined arrays of PS-*b*-PMMA cylinders with distinct and sharp boundaries between the mesa and trench regions following thermal annealing and subsequent UV irradiation of the PS films. The height difference between the DSA arrays on the mesa regions and in the trenches was measured by AFM-based depth profiling to be approximately 20 nm (Figure S2). Since the depth of the trenches covered with photo-crosslinked PS layer (B2) was 31 nm, it can be deduced that the thicknesses of the BCP film was 31 nm over the mesa regions and 41 nm in the trenches. While 10 cylinders of B1 were formed by DSA in the 380 nm-wide trench (Figure 4e; L_c_: 38 nm), nine cylinders of B2 were formed in the same size trench (L_c_, t: 42.2 nm), with eight cylinders in the 340 nm-wide mesa (L_c_, m: 42.5 nm). This increase of 4.2–4.5 nm in the L_c_ value in the case of B2 DSA may have resulted from a complementary guiding effect of DSA arrays covering both a trench and neighboring mesa region.

Hong et al. recently reported that BCP polystyrene-block-poly (ethylene oxide) (PS-*b*-PEO) forms orthogonal cylinder arrays on saw-tooth patterned sapphire substrates, with only a few nanometers difference in the top-to-valley heights [32,33]. On the basis of these results, we attempted to achieve DSA of PS-*b*-PMMA BCP with an orthogonal orientation to sub-pattern C. Here, the top-to-valley height for the PS groove pattern on the mesa was about 3 nm (Figure 5d). Following thermal annealing of the BCP film, the orthogonally aligned C1 DSA nanopattern was formed, as shown in Figure 5f. The PS groove patterns on the mesa regions played an important role in pinning the BCP cylinders and aligning them orthogonally against the grooves, but numerous defects exist at present. The uniformity of this orthogonal DSA nanopattern could be further improved by exploring the formation mechanism of the polymer nanostructure in more detail. To achieve this, the BCP film thickness would need to be controlled so as to be less than the mesa height (30 nm in this report). Nevertheless, it is clear that the uniformity of DSA and its orientation to trench patterns can be controlled through hardening the sub-layer by photo-crosslinking, optimizing the trench/mesa width in relation to the BCP equilibrium period, and careful control over surface nano-structuring.

## 3. Materials and Methods

### 3.1. Materials

Cylinder-forming polystyrene-block-poly(methyl methacrylate) (PS-*b*-PMMA) BCP with a molar mass (Mn) of PS and PMMA of 46 and 21 kg/mol, respectively, was purchased along with polydispersity (PDI; Mw/Mn of 1.09) and hydroxy-terminated polystyrene (PS-OH) (Mn: 33.5 kg/mol and PDI: 1.08) from Polymer Source, Inc. (Dorval, QC, Canada). Anhydrous toluene sourced from Aldrich (St. Louis, MO, USA) was used to solvate the block copolymer and PS-OH. The resulting polymer solution was heated to 60 °C for 1 h, and then allowed to cool to room temperature.

### 3.2. DSA Procedure

Topographic patterns consisting of trenches with three different widths (280, 380 and 530 nm) were fabricated on Si substrates using a KrF scanner (NSR-S203R, Nikon Inc., Tokyo, Japan). The depth of these trenches was ~30 nm, and their width to mesa ratio was 1:1. The surface of the Si oxide was spin-coated with PS-OH, and then thermally annealed at 150 °C for 6 h under vacuum. After washing the film with to remove any unanchored PS-OH moieties, a 16.9 nm-thick PS monolayer brush was applied. Multilayered PS-OH films were prepared without washing with toluene, and then irradiated with 22.5 J/cm^2^ of ~254 nm by UV lamp (CL 1000, UVP Inc., Upland, CA, USA) to induce full crosslinking among the polymer backbone chains [24]. A solution of ~1 wt % PS-*b*-PMMA in toluene was then spin coated onto the substrates at different speeds to control the final film thickness. For DSA pattern alignment, the coated samples were thermally annealed at 280 °C for ~100 min in a nitrogen atmosphere.

### 3.3. Characterization

The thickness of the various films was measured at each step using a film thickness measurement instrument (F20-UV, Filmetrics Inc., San Diego, CA, USA). The surface energies can be calculated from the Owens–Wendt geometric mean equation by measuring contact angles of water and diiodomethane (DM) via contact angle analyzer (DSA100, Figure S1 inset) (Krüss, Hamburg, Germany), which divides the surface free energy into the dispersive γ_s_^D^ and γ_s_^P^ terms [34]. The water and diiodomethane test liquids dropped on the PS surfaces and the contact angles were measured three times for each sample and the mean value was used. The surface topography of the various nanostructures was imaged by AFM (Dimension V, Vecco Inc., New York, NY, USA) in tapping mode. The nanostructures were not etched prior to being imaged.

## 4. Conclusions

This report has demonstrated that a photo-crosslinked PS thin film can effectively function as a sub-layer to improve the uniformity of a DSA nanopattern, which is made possible by a higher correlation length of the BCP nanostructuring layer. This is expected to prevent polymeric intermixing between the PS brush layer and BCP nanostructuring layer, thus making a hardened sub-layer likely to be an essential material requirement for the future development of DSA lithography. By precisely examining the BCP confined period by AFM, it has been confirmed that it is indeed possible to achieve defect-reduced DSA by ensuring that the trench width is a precise multiple of the BCP equilibrium period. In addition, a DSA nanopattern has been created that entirely covered the trench and mesa surfaces. Parallel or orthogonal DSA orientation can also be controlled by the thickness of the photo-crosslinked PS sub-layer.

## Figures and Tables

**Table 1:** Characteristics of PS-*b*-PMMA nanopatterns.

Sub-Layer/Sub-Pattern		L_0_ (nm)	DSA	L_c_ (nm)/Trench Width (nm)		
				280	380 Trench/Mesa	530
Si		38.6				
I/A	PS brush monolayer	41.5	A1	40.0	38.0/N.A.	37.8
II/B	photo-crosslinked PS monolayer	38.0	B1	40.0	38.0/N.A.	37.8
		38.0	B2		42.2/42.5	
III/C	photo-crosslinked PS	38.0	C1		37.5/38.4	

L_0_: equilibrium period; L_c_: confined period of DSA nanopattern in trench guide pattern.

## Supplementary Materials

The following are available online at www.mdpi.com/1996-1944/9/8/648/s1. Figure S1: Change in surface energy of photo-crosslinked PS films with increasing UV irradiation energy (wavelength: 254 nm); Figure S2: Change in thickness of photo-crosslinked PS film with initial thickness of thermally annealed PS-OH multilayer film; Figure S3: AFM image and height profile of single-layered cylinder arrays covering the whole area on the mesa and trench; Figure S4: Single-layer PS-*b*-PMMA parallel half-cylinder arrays on whole areas of mesa/trench with a 1:1 ratio.
