# Supplementary material for: Directed Self-Assembly on Photo-Crosslinked Polystyrene Sub-Layers: Nanopattern Uniformity and Orientation
Source: Materials (Basel). 2016 Aug 1;9(8):648. doi: 10.3390/ma9080648 (PMC5509098; doi:10.3390/ma9080648)
Supplement: Supplementary file 1 [file materials-09-00648-s001.pdf]

Supplementary Materials: Directed Self-Assembly on Photo-Crosslinked Polystyrene Sub-Layers: Nanopattern Uniformity and Orientation

Haeng-Deog Koh and Mi-Jeong Kim

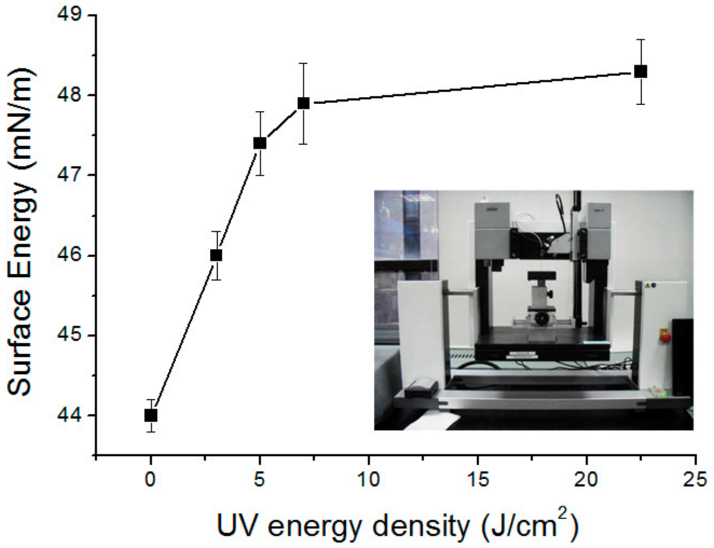

Figure S1. Change in surface energy of photo-crosslinked PS films with increasing UV irradiation energy (wavelength: 254 nm), as measured from the contact angle of two different solvents, i.e., water and diiodomethane. Inset: photo image of the contact angle analyzer instrument, DSA100.

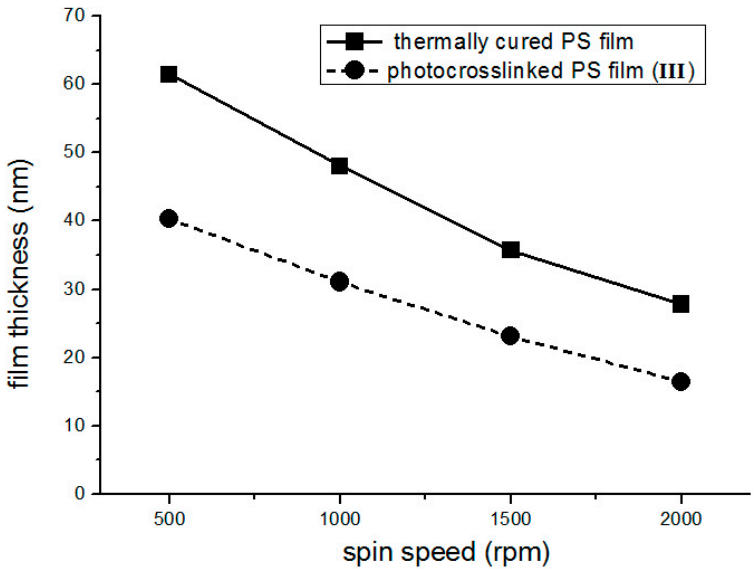

Figure S2. Change in thickness of photo-crosslinked PS film (**III**, dashed line) with initial thickness of thermally annealed PS-OH multilayer film (solid line).

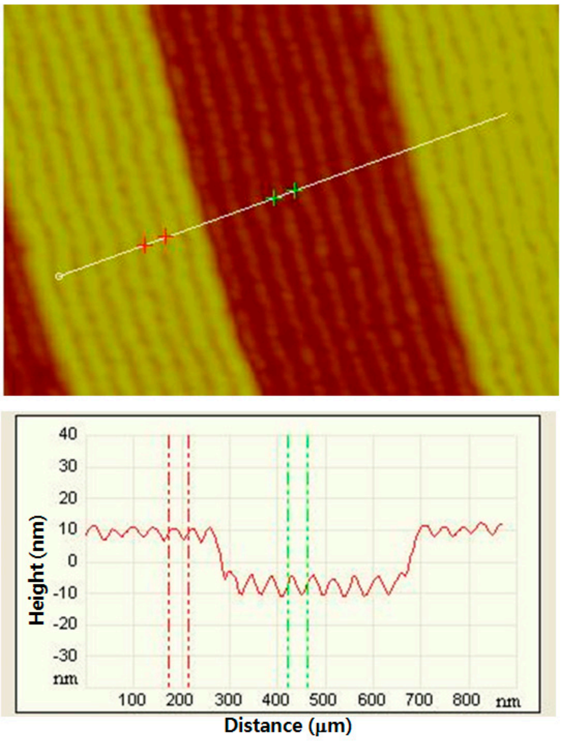

Figure S3. AFM image and height profile of single-layered cylinder arrays covering whole areas on the mesa and the trench.

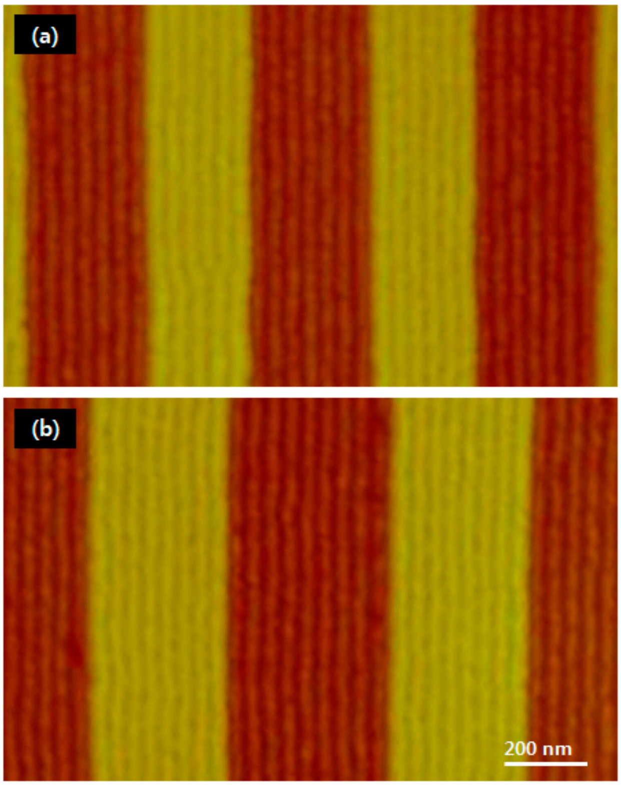

Figure S4. Single-layer PS-*b*-PMMA parallel half-cylinder arrays on whole areas of mesa/trench with a 1:1 ratio: (**a**) 280 nm and (**b**) 350 nm trench widths.
